# Antimicrobial Susceptibility and Toxin Gene Profiles of Commensal *Clostridium perfringens* Isolates from Turkeys in Hungarian Poultry Farms (2022–2023)

**DOI:** 10.3390/antibiotics14040413

**Published:** 2025-04-17

**Authors:** Ádám Kerek, Ábel Szabó, Franciska Barnácz, Bence Csirmaz, László Kovács, Ákos Jerzsele

**Affiliations:** 1 Department of Pharmacology and Toxicology, University of Veterinary Medicine, István utca 2, H-1078 Budapest, Hungary; szabo.abel@student.univet.hu (Á.S.); barnacz.franciska@student.univet.hu (F.B.); csirmaz.bence@student.univet.hu (B.C.); jerzsele.akos@univet.hu (Á.J.); 2 National Laboratory of Infectious Animal Diseases, Antimicrobial Resistance, Veterinary Public Health and Food Chain Safety, University of Veterinary Medicine, István utca 2, H-1078 Budapest, Hungary; kovacs.laszlo@univet.hu; 3 Department of Animal Hygiene, Herd Health and Mobile Clinic, University of Veterinary Medicine, István utca 2, H-1078 Budapest, Hungary; 4 Poultry-Care Kft., Lehel út 21, H-5052 Újszász, Hungary

**Keywords:** *Clostridium perfringens*, antimicrobial resistance, minimum inhibitory concentration, MIC, PCR, poultry, turkeys, Hungary

## Abstract

**Background:** The global spread of antimicrobial resistance (AMR) remains one of the greatest challenges of our time, necessitating collaboration among professionals in both the animal and public health sectors. One bacterial species that is developing AMR is *Clostridium perfringens*. It causes serious bacterial infections and continues to cause significant economic losses in the poultry industry. **Methods:** This study aimed to evaluate the antimicrobial susceptibility profiles of commensal *C. perfringens* strains isolated from large-scale turkey flocks in Hungary using minimum inhibitory concentration (MIC) determination. We complemented our research with polymerase chain reaction (PCR) analysis to detect the major and minor toxin genes that are characteristic of the species and to explore the potential associations between gene presence and antimicrobial resistance profiles. **Results:** A total of 146 commensal isolates were examined. Sensitivity to penicillin was reduced, with only 44.5% of isolates remaining susceptible, whereas 87.7% of isolates were sensitive to amoxicillin. The PCR results revealed that all isolates carried the *alpha* major toxin gene, 23.9% harbored the *beta* major toxin gene, 15.8% the *beta2* minor toxin gene, 3.4% the *NetB* minor toxin gene, and 2.7% the *epsilon* major toxin gene. No statistically significant associations were observed between the presence of toxin genes and the antimicrobial susceptibility profiles of the isolates; the MIC values showed no correlation with the presence of toxin-producing genes. **Conclusions:** *Clostridium perfringens* isolates retained susceptibility to beta-lactam antibiotics, which remain the primary choice for treatment. Regular monitoring can aid in establishing temporal trends. Future studies should include larger sample sizes and employ next-generation sequencing to further investigate multidrug-resistant strains.

## 1. Introduction

Antimicrobial resistance (AMR) is currently one of the most significant challenges facing both animal and public health. The emergence and spread of resistance restrict the use of critical antimicrobials, leaving humanity vulnerable to bacterial infections [1,2]. The exact health and economic impacts of AMR on society are difficult to quantify, due to varying perspectives and the complexity and scope of available data. However, all sources agree that the issue is serious. A 2015 report estimated that resistant pathogens infect at least 400,000 people annually in Europe, resulting in over 25,000 deaths [3]. Economically, AMR is projected to cause EUR 1.5 billion in annual losses in Europe, with EUR 900 million attributed to hospital costs alone [3,4].

Broad-spectrum oral antibiotics play a significant role in selecting and maintaining resistance genes within commensal bacteria. These genes can subsequently transfer to pathogenic bacteria, meaning that not only environmental microbes but also bacteria from a healthy gut microbiome can be potential reservoirs of antimicrobial resistance genes [5]. These resistance genes, carried by bacteria, are excreted in feces and thereby introduced into the environment. Consequently, resistant bacteria can be detected in manure, wastewater, soil, and natural water systems, eventually re-entering the food chain through feed and food, which poses obvious risks to both animal and human health [6]. However, resistomes vary significantly across different ecological conditions and exhibit only a partial overlap [7].

*C. perfringens* is a Gram-positive, anaerobic, spore-forming microbe that causes diseases in both animals and humans. It can be found in sewage, dust, and air, as well as in the intestinal tracts of healthy humans and animals [8,9,10]. It also has a remarkable proliferation rate—under optimal environmental conditions, the bacterial population can double in less than 10 min. This remarkable virulence has been attributed to the ability of *C. perfringens* to produce over 20 types of toxins and hydrolytic enzymes [9,11]. Among its toxin types, A, C, and F are associated with human infections, while B, D, E, and G are of significant veterinary importance [11,12,13,14,15]. These types are associated with various diseases: for example, enterotoxin-producing strains (CPE) are classified as type F, while strains producing the critically important virulence factor *NetB* toxin are grouped under type G, encompassing the isolates responsible for necrotic enteritis [13].

In turkeys, *C. perfringens* is implicated in necrotic enteritis, similar to its role in chickens [16,17,18,19]. However, turkeys appear to be more susceptible to cellulitis, which is caused by *C. perfringens* Type A strains, leading to greater carcass condemnation rates and processing losses compared to broilers. Additionally, cellulitis caused by Type A strains is an emerging economic concern in the United States [20,21,22,23]; in 2011, lesions associated with cellulitis made *C. perfringens* the most frequent cause of turkey meat contamination in US processing plants [24]. In addition, Type A *C. perfringens*-induced foodborne illnesses rank as the second most common bacterial foodborne infection in the US, affecting nearly one million individuals annually and resulting in net losses of approximately USD 382 million [25,26,27].

To control *C. perfringens* infections in poultry, antibiotics were previously used prophylactically, for instance as feed additives. However, the ban on prophylactic antibiotic use has led to an increase in diseases caused by *C. perfringens*, resulting in significant economic losses [28]. Excessive use of antibiotics in the past has also contributed to the widespread dissemination of AMR in *C. perfringens*. Sensitivity to tetracycline, erythromycin, and lincomycin in *C. perfringens* strains isolated from food samples has declined over time [16,29,30].

As the efficacy of antibiotics continues to decline, the importance of alternative solutions capable of replacing them is steadily increasing, prompting an ever-growing body of research in this area. Medium-chain fatty acids and triglycerides have been shown to be effective, disrupting bacterial cell wall integrity and causing damage to microbes through cytoplasmic leakage [31]. Numerous studies have demonstrated the antimicrobial properties of peptides [32], plant extracts [33], essential oils [34], and even propolis, the flavonoid content of which exhibits exceptional effectiveness [35,36]. Equally critical is the role of proper biosecurity measures and collaborative efforts with regulatory authorities [37]. Moreover, the practical application of antimicrobial therapies today necessitates sensitivity testing as a foundation for selecting appropriate treatments, supplemented by robust pharmacokinetic and pharmacodynamic investigations [38]. Considering these factors and placing increasing emphasis on alternative solutions in the future is particularly crucial; the poultry sector ranks as the second-largest consumer of antibiotics [39] after the swine industry [40]. This is especially relevant for turkey farming, where AMR-related treatment failures and disease outbreaks can result in significant productivity losses, culling costs, and food safety concerns, underscoring the urgent need for updated resistance profiles.

Given the importance of this sector and the need for continuous updates on the current situation, we aimed to assess the antimicrobial sensitivity profile of commensal *C. perfringens* strains isolated from large-scale turkey flocks in Hungary as part of our national monitoring program. Additionally, polymerase chain reaction (PCR) analyses of the isolated strains were conducted to investigate potential correlations between the presence of virulence factors and AMR profiles.

## 2. Results

### 2.1. Regional Distribution and Origin of Strains Received

A total of 146 *C. perfringens* isolates from turkeys were analyzed, and their MIC values were determined for 14 antibiotics of significant importance to animal and public health. The isolates originated from 21 large-scale turkey farms across Hungary, with 31 isolates (21.3%) from the Dél-Alföld region, 27 isolates (18.5%) from the Dél-Dunántúl region, 17 isolates (11.6%) from the Észak-Alföld region, 12 isolates (8.2%) from the Észak-Magyarország region, 15 isolates (10.3%) from the Közép-Dunántúl region, 19 isolates (13.0%) from the Közép-Magyarország region, and 25 isolates (17.1%) from the Nyugat-Dunántúl region.

### 2.2. Antimicrobial Susceptibility Testing

**Figure 1:**
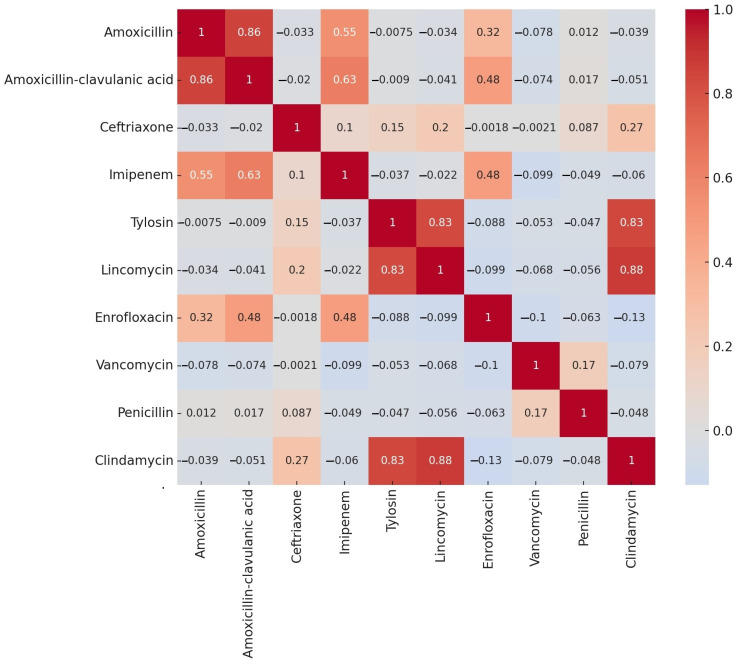
Correlation analysis of the resistance rates for each antibiotic in commensal *Clostridium perfringens* strains that were isolated from turkeys (*n* = 146), visualized using a heatmap.

We conducted correlation analyses to examine the proportions of resistant strains identified for each antibiotic based on MIC values. The results were visualized in a heatmap (Figure 1).

The correlation analysis revealed strong positive correlations between lincomycin and clindamycin (0.88), amoxicillin and amoxicillin-clavulanic acid (0.86), and tylosin and lincomycin (0.83), as well as between tylosin and clindamycin (0.83).

**Figure 2:**
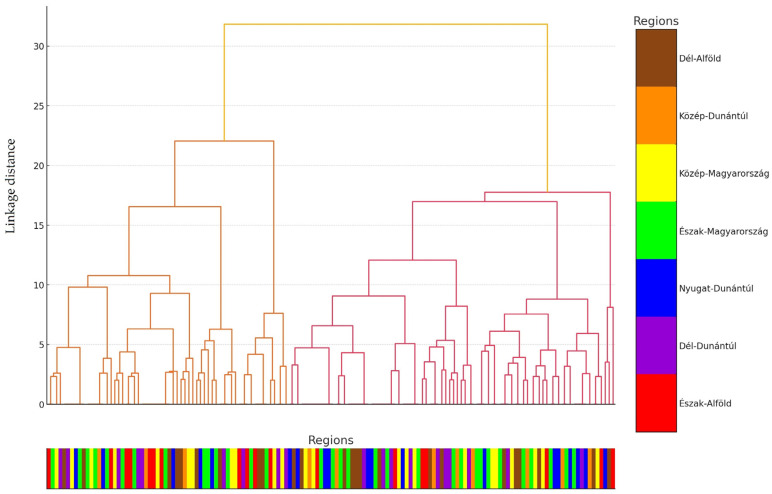
Cluster analysis of the commensal *Clostridium perfringens* strains (*n* = 146) isolated from turkeys. For clarity, individual samples are color-coded based on their regional origin, as displayed below the horizontal axis.

We also performed a hierarchical cluster analysis, visualizing the investigated strains based on their resistance profiles, which were determined by MIC values using a dendrogram (Figure 2). The clustering was based on binary-transformed MIC data across 10 antimicrobials, using Ward’s method. Each branch in the dendrogram reflects the similarity of their resistance profiles, while the colors below the dendrogram represent the regional origin of each isolate (see the Figure 2 legend).

**Figure 3:**
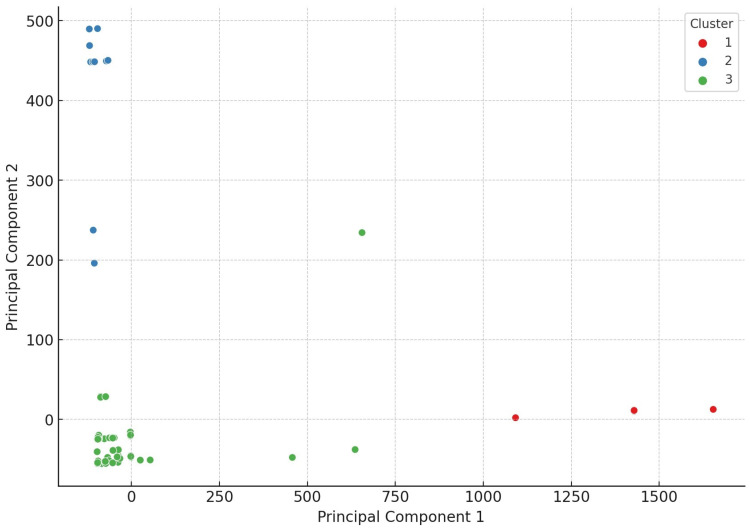
Visualization of the principal component analysis (PCA) of turkey-derived commensal *Clostridium perfringens* strains (*n* = 146), following cluster analysis. The separation of strains into three main clusters is distinctly observable.

The hierarchical cluster analysis of 146 *C perfringens* isolates was based on their MIC-derived resistance profiles against 10 antimicrobial agents. Clustering was performed using Ward’s linkage and Euclidean distance on binarized resistance data (Figure 2). Color bars beneath the dendrogram indicate the regional origin of each isolate, with the color coding defined in the right-hand legend. Based on this clustering, the isolates were assigned into three main groups. This classification supports the interpretation of Figure 3. The majority of the samples were grouped into the third and second clusters, with only a few strains being classified into the third cluster. The strains in Cluster 1 (red) exhibited exceptionally high resistance to carbapenems and lincosamides. In Cluster 2 (blue), the co-occurrence of lincosamide and fluoroquinolone resistance was predominant, whereas in Cluster 3 (green), penicillins displayed the highest resistance rates.

Notably, preliminary patterns suggest that certain clusters show partial alignment with regional origin or production type, potentially reflecting the influence of local antimicrobial usage practices or farm management strategies. These associations, however, warrant further targeted investigation.

To better visualize the distribution of isolates along a multidimensional resistance space, we performed principal component analysis (PCA) (Figure 3). In this figure, the clusters derived from hierarchical analysis are projected onto the first two principal components. The PCA does not represent a separate clustering method but, rather, illustrates the spatial separation between previously defined groups.

For variables concerning the environment and the birds themselves, significant differences were identified for amoxicillin, enrofloxacin, and clindamycin. Age groups aligned with production purposes, showing similar significant variations for the same active substances: amoxicillin (*p* = 0.0093) and clindamycin (*p* = 0.0043). Regarding farm size, significant differences were observed for enrofloxacin (*p* = 0.0499) and clindamycin (*p* = 0.0084) (Table 1).

The frequency tables present the occurrence rates and percentages of MIC values, along with the calculated MIC_50_ and MIC_90_ values for the tested antibiotics within the studied population. Among the substances with established clinical breakpoints, both the MIC_50_ and MIC_90_ values were below the clinical breakpoint for imipenem. However, for amoxicillin and clindamycin, only the MIC_50_ value fell below the clinical breakpoint. An official epidemiological cut-off (ECOFF) value was available solely for clindamycin (0.125 µg/mL), yet the calculated MIC_50_ and MIC_90_ values were significantly higher (Table 2).

A complete dataset summarizing the antimicrobial resistance phenotypes, geographic origin, and PCR-based toxin gene profiles of all isolates is available in the Supplementary Materials.

**Figure 4:**
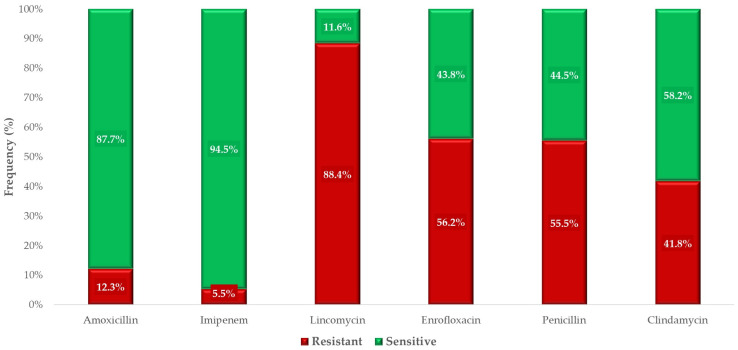
Ocular susceptibility profile of *Clostridium perfringens* strains of turkey origin (*n* = 146) to antibiotic agents with clinical breakpoints.

For antibiotics with established clinical breakpoints, we determined the phenotypic clinical susceptibility of the examined population (Figure 4). A significant proportion of the isolates retained susceptibility to amoxicillin (87.7%), one of the primary choices for treating *C. perfringens*-associated infections. However, sensitivity to penicillin was markedly reduced, with only 44.5% of isolates being susceptible. Imipenem, a critically important antibiotic for treating human multidrug-resistant infections, exhibited 94.5% susceptibility. The highest level of resistance was observed against lincomycin, with only 11.6% of isolates demonstrating susceptibility.

**Figure 5:**
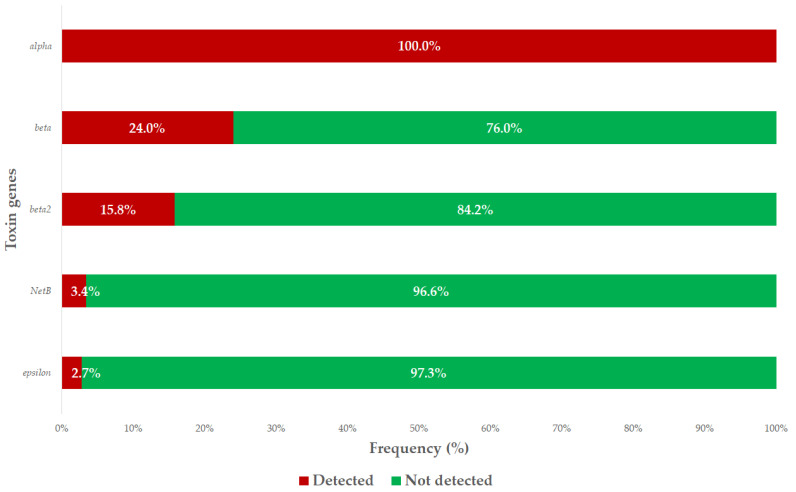
Prevalence of the major and minor toxin genes among *Clostridium perfringens* isolates (*n* = 146). The presence or absence of each gene was determined by real-time PCR. “Detected” indicates a positive PCR result for the corresponding gene.

We conducted PCR analysis of the isolates to identify those genes responsible for major and minor toxin production. All examined isolates tested positive for the major *alpha* toxin gene, which is universally present in *C. perfringens* strains. Among the isolates, 23.9% were positive for the major *beta* toxin gene, 2.7% for the major *epsilon* toxin gene, 15.8% for the minor *beta2* toxin gene, and 3.4% for the minor *NetB* toxin gene. No isolates tested positive for the major *iota* toxin or the minor *enterotoxin* genes. The prevalence of each toxin gene is summarized in Figure 5, providing a visual overview of the virulence-associated genetic potential across the tested isolates.

When examining the co-occurrence of multiple toxin genes within individual isolates (excluding the universally present major *alpha* gene), we observed the following: the highest number of simultaneous toxin genes (major *beta*, *epsilon*, minor *beta2*, and minor *NetB*) occurred in a single isolate. The combination of major *beta*, minor *beta2*, and minor *NetB* genes was detected in 4 isolates; major *beta*, major *epsilon*, and minor *beta2* genes co-occurred in 3 isolates; major *beta* and minor *beta2* genes were found together in 10 isolates; and major *beta* and major *epsilon* genes co-occurred in 1 isolate.

We also investigated potential associations between the presence of specific toxin genes and the MIC values for each antibiotic. However, no statistically significant differences in MIC values were observed between isolates carrying specific toxin genes and those without these genes for any of the antibiotics tested.

## 3. Discussion

In this study, we determined the MIC values of 146 *C. perfringens* strains isolated from turkeys for antibiotics of public and veterinary health significance. To complement our findings, we conducted PCR analyses to identify the presence of major and minor toxin-producing genes and to explore potential correlations between antimicrobial resistance patterns and the presence of these toxin genes.

Regarding beta-lactam antibiotics, our study on commensal *C. perfringens* strains (*n* = 146) revealed a 55.5% resistance rate to penicillin, with MIC_50_ and MIC_90_ values of 1 µg/mL and 32 µg/mL, respectively. In contrast, Slavic et al. did not identify any penicillin-resistant strains, reporting MIC_50_ and MIC_90_ values of 0.06 µg/mL and 0.12 µg/mL, respectively [16]. Similarly, Silva et al. found no penicillin-resistant strains in isolates from broiler chickens, with MIC values not exceeding 0.25 µg/mL [41]. For amoxicillin, we observed 12.3% resistance, whereas Martel et al. found no resistant strains in broiler-derived isolates, and Osman and Elhariri reported a 7% resistance rate [42,43]. Regarding imipenem, our study demonstrated a 5.5% resistance rate, while Akhi et al. reported a significantly higher resistance rate of 38% in *C. perfringens* strains isolated from human fecal samples. Akhi et al. documented MIC values reaching as high as 32 µg/mL, whereas the highest MIC value we observed for imipenem was 16 µg/mL [44]. For ceftriaxone, Akhi et al. reported MIC values ranging from 0.016 to 128 µg/mL, while our study identified strains with MIC values as high as 256 µg/mL [44].

These findings are consistent with the global AMR trends in *C. perfringens* reported in various regions, including Asia, North America, and the Middle East, where increasing resistance to beta-lactams, fluoroquinolones, and lincosamides has been documented. This underscores the widespread and evolving nature of AMR in both food-producing animals and the environment [45,46,47,48].

Penicillins are considered first-line antibiotics for treating infections caused by *C. perfringens*. The observed differences in susceptibility can likely be attributed to variations in antibiotic usage patterns and the overuse of these agents over several decades. Overall, our findings indicate a significant decrease in sensitivity to penicillin, while amoxicillin has largely retained its efficacy. This reduction in effectiveness is an undesirable trend, highlighting the importance of regular monitoring studies. Imipenem, categorized as a lifesaving “last resort” antibiotic, holds critical significance for human health, warranting constant surveillance. Although the observed resistance rate of 5.5% may seem low, the presence of any resistant strains at all is concerning. However, this figure is more likely to be a result of the instability of the antibiotic’s aqueous solution rather than true resistance [49]. For amoxicillin-clavulanic acid, we recorded MIC_50_ and MIC_90_ values of 0.5 µg/mL and 8 µg/mL, respectively. Comparative data specific to poultry are lacking; however, Park et al. reported that 84% of strains isolated from horses were susceptible to amoxicillin-clavulanic acid [50]. Since *C. perfringens* strains are generally not known to produce beta-lactamase enzymes, the use of clavulanic acid—already unauthorized for poultry—is not justified in practice for treating *C. perfringens* infections.

Among the lincosamides, only 11.6% of the strains we tested were sensitive to lincomycin, which contrasts sharply with the findings of Silva et al., who reported that 89.1% of their isolates were sensitive to this antibiotic. While Silva et al. did not observe MIC values exceeding 64 µg/mL, we recorded MIC values as high as 1024 µg/mL [41]. Gholamiandeh-Kordi et al. and Martel et al. reported resistance rates of 61.5% and 63.3%, respectively, which are closer to our observed resistance rate of 89.1% [46,47]. In contrast, Osman and Elhariri did not identify any lincomycin-sensitive strains [43]. For clindamycin, another antibiotic in this group, we observed a resistance rate of 41.8%, with MIC_50_ and MIC_90_ values of 2 µg/mL and 32 µg/mL, respectively. Slavic et al., however, reported a resistance rate of only 2%, with MIC_50_ and MIC_90_ values of 1 µg/mL and 4 µg/mL, respectively [16].

The high resistance rates that we observed for clindamycin are concerning, particularly given its broad spectrum of activity. Since clindamycin is primarily used in companion animals and human medicine, its importance is founded mainly on in vitro studies and in the context of cross-resistance with lincomycin.

For the nitroimidazole antibiotic metronidazole, the MIC_50_ and MIC_90_ values of the strains we tested were 32 µg/mL and 512 µg/mL, respectively. In contrast, Slavic et al. reported much lower MIC values of 1 µg/mL (MIC_50_) and 4 µg/mL (MIC_90_) [16]. For ronidazole, we observed MIC_50_ and MIC_90_ values of 32 µg/mL and 256 µg/mL, respectively, whereas Dutta et al. found no strains with MIC values exceeding 0.5 µg/mL in bird-derived isolates [51]. The relevance of nitroimidazoles lies primarily in research contexts, as their use is prohibited in food-producing animals.

Regarding enrofloxacin, the results differ widely. In this study, 56.2% of the strains were resistant. In human cases, second-generation fluoroquinolones, such as enrofloxacin, are effective against anaerobic bacteria like *C. perfringens* in only about half of the cases, which fits with our results. However, in 2013, Osman and Elhariri reported an 82% resistance rate to enrofloxacin among isolates from broiler chickens in Egypt [43]. In comparison, a 2022 study found that 98% of the *C. perfringens* strains tested were sensitive to enrofloxacin [52]. These notable differences in resistance suggest that AMR in *C. perfringens* is constantly developing in isolated colonies, which leads to varying levels of resistance. Second-generation fluoroquinolones, such as enrofloxacin, are effective against anaerobic bacteria like *C. perfringens* in only about half of the cases [53]. This limited efficacy likely explains the high resistance rates observed. Consequently, the use of these antibiotics should be reserved for complicated infections where a broad-spectrum agent is necessary.

For turkey-derived strains of *C. perfringens*, Watkins et al. reported MIC_50_ and MIC_90_ values of 2 µg/mL and 256 µg/mL, respectively, for tylosin and tilmicosin [54]. In comparison, our study found MIC_50_ values of 1 µg/mL for tylosin and 16 µg/mL for tilmicosin, with MIC_90_ values of 64 µg/mL for both antibiotics. Although the MIC_50_ values we calculated are more favorable on a population level, the less favorable MIC_90_ values indicate potential challenges in population management. The primary limitation of this analysis lies in the relatively small sample size, which may have influenced the findings. However, discrepancies with other studies may also stem from regional differences in antimicrobial usage practices, animal husbandry systems, biosecurity measures, and the genetic variability of circulating *C. perfringens* strains—all of which warrant further comparative investigation. Future studies incorporating larger sample sizes could provide a more accurate assessment.

Additionally, the overuse of tetracyclines over the decades has significantly impacted their effectiveness, as evidenced by the resistance patterns observed. For oxytetracycline, we observed MIC_50_ and MIC_90_ values of 16 µg/mL and 32 µg/mL, respectively, while Gharaibeh et al. reported 0.5 µg/mL for MIC_50_ and 256 µg/mL for MIC_90_ [55]. This broad range of MIC values is likely attributable to differences in sample sizes, highlighting the need for larger-scale, standardized studies.

Vancomycin is now exclusively used in public health settings for the treatment of life-threatening infections caused by Gram-positive bacteria, and resistance to this drug may indicate a troubling trend. In their experiment, Johansson et al. did not report MIC values exceeding 4 µg/mL [30], whereas our study found MIC_50_ and MIC_90_ values of 1 µg/mL and 32 µg/mL, respectively. Although there are no clinical breakpoints for the veterinary use of vancomycin, due to its critical importance in human medicine, the higher MIC in our study is concerning as it suggests that *C. perfringens* is developing resistance to even little-used antibiotics. As vancomycin is not approved for veterinary use, the turkeys will not have come into contact with it. Therefore, the observed resistance may be a result of genetic transfer. Next-generation sequencing would shed more light on the mechanisms driving resistance development in this case. Due to its critical importance in human medicine, no clinical breakpoints are available for veterinary use, further highlighting the need for careful stewardship.

The detection of elevated vancomycin MICs in commensal *C. perfringens* strains from turkeys raises important concerns, even though this antibiotic is not used in poultry. The presence of resistance may be due to horizontal gene transfer via plasmids or transposons, as demonstrated in other Gram-positive species such as *Enterococcus* and *Staphylococcus*. If such resistance elements are mobile, they may spread within the poultry microbiota or could even cross species barriers. From a food safety and One Health perspective, this could pose a significant risk, especially if resistant strains enter the human food chain. This further underlines the need for genomic surveillance and prudent antimicrobial stewardship, even in cases where antibiotics are not directly applied in animal production [56,57,58].

In our study, we examined the presence of major and minor toxin genes in 146 commensal *C. perfringens* isolates, including those encoding major *alpha*, *beta*, *epsilon*, and *iota* toxins, as well as minor *beta2*, *enterotoxin*, and *NetB* toxins. The alpha toxin gene, as expected, was present in all isolates, consistent with its universal expression in *C. perfringens*. Additionally, 3.4% of isolates carried the minor *NetB* toxin gene. Lyhs et al. investigated 212 *C. perfringens* isolates, of which 174 were derived from diseased turkeys and 38 from healthy individuals. Similar to our findings, Lyhs et al. detected the *alpha* toxin gene in all isolates. However, unlike our results, they reported the presence of the *NetB* toxin gene exclusively in isolates from turkeys suffering from necrotic enteritis, with a prevalence of 8%, and did not detect this gene in commensal isolates [59]. In our study, 15.8% of isolates carried the *beta2* toxin gene, whereas Lyhs et al. found this in only one isolate [59]. Additionally, Lyhs et al. did not detect the genes encoding major *beta*, *epsilon*, or *iota* toxins in their isolates, whereas we found these genes in 23.9%, 2.7%, and 0% of our isolates, respectively. We also did not detect the minor *enterotoxin* gene in any isolate, which is consistent with the findings of Mohiuddin et al., who observed the *NetB* toxin gene in two poultry-derived isolates but did not detect the genes encoding major *beta*, *epsilon*, *iota*, or minor enterotoxins [28]. Slavic et al. identified the *epsilon* toxin gene in one turkey-derived isolate, which also harbored the *beta* toxin gene. Furthermore, they found the minor *beta2* toxin gene in 90% of turkey-derived isolates. Although we did not observe statistically significant associations between the presence of virulence genes and resistance profiles, Slavic et al. reported that *C. perfringens* isolates that were resistant to clindamycin and erythromycin derived from swine were significantly less likely to carry the *beta2* toxin gene. However, no significant correlations between antibiotic susceptibility and toxin gene presence were found for isolates from cattle, chickens, or turkeys [16]. While our study did not reveal significant differences between antibiotic susceptibility and the presence of toxin genes, Wei et al. reported significant differences for clindamycin among *C. perfringens* isolates derived from healthy chickens and from those with necrotic enteritis. The isolates from diseased chickens exhibited lower susceptibility to clindamycin and gentamicin [60].

The detection of virulence-associated genes in the isolates from turkeys in Hungary may have important implications for local poultry health. Although our study focused on apparently healthy birds, the presence of these toxin genes—especially in antimicrobial-resistant strains—suggests that subclinical reservoirs of potentially pathogenic *C. perfringens* are currently circulating within flocks. These strains could act as a source of infection under stress or immunosuppressive conditions, thereby increasing the risk of necrotic enteritis outbreaks. This highlights the importance of integrating toxin gene monitoring into AMR surveillance frameworks, particularly in countries like Hungary with intensive turkey production systems.

Based on the findings reported in the literature, it can be hypothesized that there are significant differences in the relationship between the presence of virulence factors and resistance profiles. One key limitation of our study was the sample size, which likely constrained our ability to detect such associations. Although our analyses did not reveal any significant differences linked to the presence or absence of virulence genes, the correlations reported in previous studies emphasize the importance of continuing investigations with larger sample sizes in the future.

Nonetheless, certain non-significant trends in our dataset, such as slightly higher resistance rates among *beta2*-positive isolates, may suggest a biological interplay between toxin gene carriage and resistance mechanisms. It is plausible that mobile genetic elements harboring both toxin and resistance determinants contribute to such a co-occurrence, as seen in other *Clostridium* species. These potential links merit further exploration using genomic approaches such as plasmid or transposon mapping.

The limitations of this study include its moderate sample size, potential sampling bias due to the opportunistic nature of strain collection from specific farms, and the cross-sectional design. Longitudinal monitoring would be necessary to track temporal resistance trends and to establish causal relationships between antimicrobial use and resistance emergence.

From a practical perspective, the resistance patterns observed in this study highlight the pressing need for improved antimicrobial stewardship in turkey production. Regular monitoring of resistance profiles at the farm level, along with regionally tailored treatment protocols, can help preserve antibiotic efficacy. Furthermore, the integration of non-antibiotic alternatives—such as competitive exclusion products, essential oils, or vaccination—should be encouraged as part of a holistic AMR management strategy. Promising non-antibiotic interventions—such as medium-chain fatty acids, essential oils, competitive exclusion cultures, and bacteriophage therapy—may offer effective preventive options, particularly when integrated with enhanced biosecurity and vaccination programs.

## 4. Materials and Methods

### 4.1. The Origin of the Strains

The examined strains were collected between February 2022 and May 2023 as part of routine diagnostic sampling conducted by veterinarians on large-scale livestock farms. For each sample, information was collected regarding the organ of origin (e.g., the trachea or cloaca) and the geographic location (the specific municipality). Based on the municipalities, the samples were categorized into one of Hungary’s seven administrative regions.

Selective isolation of the strains was performed using CHROMagar™ *C. perfringens* (Chebio Fejlesztő Kft., Budapest, Hungary), and anaerobic cultivation and testing were carried out using the BD GasPak™ system (VWR International Kft., Debrecen, Hungary). Pure cultures were prepared on tryptone soy agar (Biolab Zrt., Budapest, Hungary) and subsequently preserved using the Microbank™ system (Pro-Lab Diagnostics, Richmond Hill, ON, Canada) at −80 °C.

Based on the municipalities, the samples were categorized into one of Hungary’s seven administrative regions. This regional classification allows for the assessment of potential geographic patterns in antimicrobial resistance, although uneven sample distribution may influence the representativeness of certain regions.

### 4.2. Minimum Inhibitory Concentration (MIC) Determination

The phenotypic expression of AMR was assessed by determining the MIC values for each bacterial strain. The methodology followed the guidelines of the Clinical Laboratory Standard Institute (CLSI) [61], with breakpoints established according to the CLSI recommendations [62]. Where available, the results were also compared with the ECOFF values defined by the European Committee on Antimicrobial Susceptibility Testing (EUCAST).

Bacterial strains stored at −80 °C were resuspended in 3 mL of cation-adjusted Müller–Hinton broth (CAMHB) the day before testing and incubated at 37 °C for 18–24 h. The MIC testing was performed using 96-well microtiter plates (VWR International, LLC., Debrecen, Hungary). Except for the first column, all wells were filled with 90 µL of CAMHB. Stock solutions of the tested antimicrobial agents (Merck KGaA, Darmstadt, Germany) were prepared at 1024 µg/mL according to CLSI guidelines [63]. From the 512 µg/mL half-diluted solution, 180 µL was transferred into the first column of the microtiter plates, and a twofold serial dilution was performed across the plate. After the 10th column, 90 µL of excess solution was discarded, leaving 90 µL in each well. Using a nephelometer (ThermoFisher Scientific, Budapest, Hungary), bacterial suspensions were adjusted to a turbidity of 0.5 McFarland and inoculated into the microtiter plates starting from the 11th column, with 10 µL added per well [63]. The MIC values were determined using the Sensititre™ SWIN™ automatic MIC reader (ThermoFisher Scientific, Budapest, Hungary) and analyzed with the VIZION system software, version 3.4 (ThermoFisher Scientific, Budapest, Hungary, 2024). The reference quality control strain used was *C. perfringens* (ATCC 13124).

### 4.3. PCR Tests

We performed PCR analysis to map the major and minor toxin gene profiles of each strain. DNA extraction from bacterial suspensions was carried out using the Zymo Quick-DNA Fungal/Bacterial Miniprep Kit (Zymo Research, Murphy Ave., Irvine, CA, USA) following the manufacturer’s instructions. For bead-beating to release bacterial genetic material, we used the Qiagen TissueLyzer LT (Qiagen GmBH, Hilden, Germany) at 50 Hz for 5 min.

For PCR testing, we utilized the Kylt *Clostridium perfringens* real-time PCR kit (Kylt, Höltinghausen, Germany), which is designed for the detection of major (*alpha*, *beta*, *epsilon*, and *iota*) and minor (*b2*, *entero*, and *NetB*) toxin genes in avian samples. The kit comprises a dual multiplex real-time PCR system containing all necessary reagents, including primers, probes, positive and negative controls, and an internal amplification control, ensuring standardized detection and internal quality control. All procedures followed the manufacturer’s protocol, and the extracted nucleic acids were stored at −20 °C until use.

PCR analyses were conducted using the CFX Opus Dx real-time PCR system (Bio-Rad Hungary Ltd., Budapest, Hungary). The results were evaluated using the Bio-Rad CFX Maestro software, version 5.3.022.1030 (Bio-Rad Hungary Ltd., Budapest, Hungary).

Amplification was performed in a total volume of 20 µL, including 18 µL of Kylt Reaction-Mix (Multiplex 1 or 2) and 2 µL of sample DNA. The thermal cycling protocol consisted of initial enzyme activation at 95 °C for 10 min, followed by 42 cycles of denaturation at 95 °C for 15 s and annealing/extension at 60 °C for 60 s. Fluorescence was measured at each cycle using the FAM, Cy5, TXR, and HEX channels. Each run included positive controls for both multiplex reactions and a negative control, in accordance with the manufacturer’s instructions.

The presence of the *alpha* gene served as an internal amplification control, as it is universally present in all *C. perfringens* strains. Reactions were only deemed valid if all control curves behaved according to protocol-defined expectations.

### 4.4. Statistic Analysis

Analysis of the data was performed using R version 4.1.0 [64]. The normality of data distribution was assessed using the Shapiro–Wilk test [65]. For datasets that did not conform to normal distribution assumptions, non-parametric statistical methods were applied. Specifically, the Kruskal–Wallis test [66] was used to evaluate resistance levels across multiple sample groups. This non-parametric approach is particularly suited for comparing medians among groups without assuming normality. To pinpoint specific differences between the groups, post hoc analyses were conducted using pairwise comparisons via the Mann–Whitney U-test [67] and *t*-tests, where appropriate [68]. To mitigate the risk of Type I errors caused by multiple comparisons, Bonferroni correction was applied [69], recognizing that this method may increase the likelihood of Type II errors (overlooking true differences).

To explore the relationships among active substances, correlation analysis was employed. Additionally, PCA [70] was conducted to uncover underlying patterns and to visualize similarities or differences within the data. PCA was used to reduce the dimensionality of the dataset while retaining as much variance as possible. This approach is particularly useful for datasets with numerous variables, which can complicate analysis and visualization. PCA transforms the data into new variables called principal components, with the first component accounting for the largest variance in the dataset. Subsequent components explain progressively smaller amounts of variance. These components are orthogonal, meaning that they are uncorrelated with one another, thereby simplifying the interpretation of complex datasets.

Hierarchical cluster analysis followed, with the results presented as a dendrogram [71]. This visual representation effectively illustrates the clustering of isolates and the hierarchical relationships among them. Cluster analysis is a method for identifying patterns in data by grouping similar observations into clusters. Each cluster contains data points that share common characteristics, distinct from those in other clusters. Hierarchical clustering results were depicted using a dendrogram, a tree-like diagram illustrating the hierarchy and relationships among clusters.

The use of PCA prior to clustering served two purposes: to reduce dimensionality while preserving major variance components, and to eliminate multicollinearity among antimicrobial variables. Hierarchical clustering was performed on the principal component scores using Euclidean distance and Ward’s method, which ensures compact, interpretable clusters based on overall similarity patterns. This combined approach facilitates robust classification while minimizing noise from irrelevant variables.

Correlation analysis focuses on assessing the strength and direction of relationships between variables. It evaluates how changes in one variable correspond to changes in another. A positive correlation implies that as one variable increases, the other also increases, whereas a negative correlation indicates an inverse relationship. The strength of the correlation is quantified by the correlation coefficient, ranging from −1 to +1. A coefficient of +1 denotes a perfect positive correlation, −1 represents a perfect negative correlation, and 0 indicates no linear relationship.

## 5. Conclusions

Overall, our findings underscore the critical importance of the continuous monitoring of AMR. While our results were consistent with previous studies in several cases, notable discrepancies were observed. These variations are likely attributable to the low sample size in our study. However, other factors such as country-specific antibiotic usage practices, differences in flock sizes, variations in the antibiotics administered, and treatment durations during rearing may also play significant roles.

As for enrofloxacin, the varying levels of resistance to this antibiotic are likely to be the result of isolated developments. This is concerning, as the inconsistency between strains will make *C. perfringens* harder to treat. Consequently, to prevent resistance developing further, the use of enrofloxacin should be reserved for complicated infections where a broad-spectrum agent is necessary.

Based on these observations, future studies should aim to increase sample sizes significantly and include isolates derived from clinical cases alongside commensal strains. The inclusion of clinical strains could reveal more pronounced differences in resistance patterns, particularly concerning the presence of virulence-associated genes. Furthermore, multidrug-resistant strains should be subjected to next-generation sequencing to comprehensively explore the genetic mechanisms underlying phenotypic resistance. This approach could provide a deeper understanding of resistance patterns and contribute to the development of targeted interventions.

Incorporating clinical isolates in addition to commensal strains could provide particularly valuable insights. Comparing these datasets with an adequate sample size would likely yield even more meaningful results, enhancing our understanding of the complex interplay between virulence factors and antimicrobial resistance.

In addition to scientific insights, these findings carry important implications for policy and on-farm decision-making. Regulatory bodies should support the development of national AMR surveillance frameworks tailored to species and regional profiles, while restricting the use of critically important antibiotics, such as fluoroquinolones, in preventive protocols. Promoting antimicrobial stewardship training and incentivizing the adoption of non-antibiotic alternatives—such as probiotics, vaccines, or feed-based interventions—may also play a pivotal role in reducing selection pressure and safeguarding antimicrobial efficacy.

## Figures and Tables

**Table 1:** Statistical comparison of the resistance profiles of the samples according to utilization direction, age group, and plant size.

Antibiotics	Broiler–Breeding	^1^ Young–^2^ Adult	^3^ Small–^4^ Medium
Amoxicillin	0.0093 *	0.0093 *	0.0915
Imipenem	0.0973	0.0973	0.5595
Lincomycin	0.6313	0.6313	0.7438
Enrofloxacin	0.4941	0.4941	0.0499 *
Penicillin	0.6939	0.6939	0.8216
Clindamycin	0.0043 *	0.0043 *	0.0084 *

* Significant difference (*p* < 0.05); ^1^ younger than 13 weeks; ^2^ older than 13 weeks; ^3^ small (5001–50,000); ^4^ medium (50,001–100,000).

**Table 2:** The frequency table of minimum inhibitory concentration (MIC) values obtained for the tested antibiotics with established clinical breakpoints in turkey-derived *Clostridium perfringens* isolates (*n* = 146). For each antibiotic, the upper row indicates the number of isolates, while the lower row represents the corresponding percentage. The vertical red line indicates the clinical breakpoints.

Antibiotic	^1^ BP *	0.001	0.002	0.004	0.008	0.016	0.03	0.06	0.125	0.25	0.5	1	2	4	8	16	32	64	128	256	512	1024	MIC_50_	MIC_90_	^2^ ECOFF
	µg/mL																						µg/mL		
Amoxicillin	16	1	0	5	6	9	6	8	19	18	26	15	12	3	0	0	18						0.5	32	-
		0.7%	0.0%	3.4%	4.1%	6.2%	4.1%	5.5%	13.0%	12.3%	17.8%	10.3%	8.2%	2.1%	0.0%	0.0%	12.3%								
Imipenem	16	4	0	0	3	5	21	10	14	13	16	10	18	3	21	8							0.5	8	-
		2.7%	0.0%	0.0%	2.1%	3.4%	14.4%	6.8%	9.6%	8.9%	11.0%	6.8%	12.3%	2.1%	14.5%	5.5%									
Lincomycin	1				3	0	0	0	2	1	11	5	32	11	6	8	10	46	2	0	4	5	16	64	-
					2.1%	0.0%	0.0%	0.0%	1.4%	0.7%	7.5%	3.4%	21.9%	7.5%	4.1%	5.5%	6.8%	31.5%	1.4%	0.0%	2.7%	3.4%			
Enrofloxacin	2					1	0	0	6	14	15	28	23	9	12	38							2	16	-
						0.7%	0.0%	0.0%	4.1%	9.6%	10.3%	19.2%	15.8%	6.2%	8.2%	26.0%									
Penicillin	1				1	3	5	4	6	20	26	28	18	8	5	2	13	0	1	3	3		1	32	-
					0.7%	2.1%	3.4%	2.7%	4.1%	13.7%	17.8%	19.2%	12.3%	5.5%	3.4%	1.4%	8.9%	0.0%	0.7%	2.1%	2.1%				
Clindamycin	8			1	16	1	3	12	8	12	8	5	15	4	5	6	39	1	2	1	6	1	2	32	0.125
				0.7%	11.0%	0.7%	2.1%	8.2%	5.5%	8.2%	5.5%	3.4%	10.3%	2.7%	3.4%	4.1%	26.7%	0.7%	1.4%	0.7%	4.1%	0.7%			

* BP—breakpoint; ^1^ Clinical Laboratory Standard Institute (CLSI); ^2^ Epidemiological cut-off value given by the European Committee on Antimicrobial Susceptibility Testing (EUCAST).

## Data Availability

The data presented in this study are available from the corresponding author upon reasonable request.

## Supplementary Materials

The following supporting information can be downloaded at: https://www.mdpi.com/article/10.3390/antibiotics14040413/s1. Table S1: Frequency table of the minimum inhibitory concentration (MIC) values (µg/mL) for agents without breakpoints in *Clostridium perfringens* samples (*n*=146) derived from turkeys. Table S2. Additional data.
