# Supplementary material for: Antimicrobial Susceptibility and Toxin Gene Profiles of Commensal Clostridium perfringens Isolates from Turkeys in Hungarian Poultry Farms (2022–2023)
Source: Antibiotics (Basel). 2025 Apr 17;14(4):413. doi: 10.3390/antibiotics14040413 (PMC12024083; doi:10.3390/antibiotics14040413)
Supplement: Supplementary file 1 [file antibiotics-14-00413-s001.zip › Table S1.pdf]

**Supplementary Table S1.** Frequency table of the minimum inhibitory concentration (MIC) values (µg/mL) for agents without breakpoints in *Clostridium perfringens* samples (*n*=146) derived from turkeys. The top row for each agent shows the count, while the bottom row shows the percentage.

| Antibiotic | 0.001 | 0.002 | 0.004 | 0.008 | 0.016 | 0.03 | 0.06 | 0.125 | 0.25 | 0.5 | 1 | 2 | 4 | 8 | 16 | 32 | 64 | 128 | 256 | 512 | 1024 | MIC$_{50}$ | MIC$_{90}$ |
|---|---|---|---|---|---|---|---|---|---|---|---|---|---|---|---|---|---|---|---|---|---|---|---|
| | | | | | | | | | | | | | | | | | | | | | | µg/mL | |
| *Amoxicillin-clavulanic acid | 1 | 1 | 1 | 9 | 9 | 6 | 13 | 17 | 13 | 30 | 13 | 12 | 5 | 2 | 3 | 11 | | | | | | 0.5 | 8 |
| | 0.7% | 0.7% | 0.7% | 6.2% | 6.2% | 4.1% | 8.9% | 11.6% | 8.9% | 20.5% | 8.9% | 8.2% | 3.4% | 1.4% | 2.1% | 7.5% | | | | | | | |
| Ceftriaxone | 1 | 4 | 2 | 5 | 2 | 10 | 8 | 6 | 11 | 17 | 24 | 11 | 9 | 6 | 18 | 4 | 2 | 2 | 4 | | | 1 | 16 |
| | 0.7% | 2.7% | 1.4% | 3.4% | 1.4% | 6.8% | 5.5% | 4.1% | 7.5% | 11.6% | 16.4% | 7.5% | 6.2% | 4.1% | 12.3% | 2.7% | 1.4% | 1.4% | 2.7% | | | | |
| Tylosin | | | | 3 | 3 | 9 | 9 | 31 | 8 | 5 | 21 | 15 | 4 | 2 | 2 | 2 | 26 | 0 | 0 | 1 | 5 | 1 | 64 |
| | | | | 2.1% | 2.1% | 6.2% | 6.2% | 21.2% | 5.5% | 3.4% | 14.4% | 10.3% | 2.7% | 1.4% | 1.4% | 1.4% | 17.8% | 0.0% | 0.0% | 0.7% | 3.4% | | |
| Vancomycin | | | | 1 | 1 | 0 | 1 | 20 | 6 | 13 | 33 | 7 | 4 | 5 | 3 | 39 | 0 | 0 | 3 | 10 | | 1 | 32 |
| | | | | 0.7% | 0.7% | 0.0% | 0.7% | 13.7% | 4.1% | 8.9% | 22.6% | 4.8% | 2.7% | 3.4% | 2.1% | 26.7% | 0.0% | 0.0% | 2.1% | 6.8% | | | |
| Oxytetracycline | | | | | | 3 | 4 | 6 | 0 | 3 | 10 | 6 | 21 | 15 | 12 | 52 | 0 | 2 | 6 | 5 | 1 | 16 | 32 |
| | | | | | | 2.1% | 2.7% | 4.1% | 0.0% | 2.1% | 6.8% | 4.1% | 14.4% | 10.3% | 8.2% | 35.6% | 0.0% | 1.4% | 4.1% | 3.4% | 0.7% | | |
| Tilmicosin | 2 | 0 | 1 | 0 | 1 | 2 | 2 | 7 | 5 | 7 | 13 | 11 | 4 | 14 | 16 | 18 | 34 | 0 | 2 | 2 | 5 | 16 | 64 |
| | 1.4% | 0.0% | 0.7% | 0.0% | 0.7% | 1.4% | 1.4% | 4.8% | 3.4% | 4.8% | 8.9% | 7.5% | 2.7% | 9.6% | 11.0% | 12.3% | 23.3% | 0.0% | 1.4% | 1.4% | 3.4% | | |
| Ronidazole | | | | | 1 | 1 | 1 | 4 | 7 | 16 | 11 | 19 | 0 | 0 | 1 | 58 | 3 | 4 | 7 | 12 | 1 | 32 | 256 |
| | | | | | 0.7% | 0.7% | 0.7% | 2.7% | 4.8% | 11.0% | 7.5% | 13.0% | 0.0% | 0.0% | 0.7% | 39.7% | 2.1% | 2.7% | 4.8% | 8.2% | 0.7% | | |
| Metronidazole | | | | 1 | 0 | 0 | 3 | 5 | 2 | 9 | 12 | 5 | 0 | 5 | 6 | 61 | 0 | 2 | 12 | 20 | 3 | 32 | 512 |
| | | | | 0.7% | 0.0% | 0.0% | 2.1% | 3.4% | 1.4% | 6.2% | 8.2% | 3.4% | 0.0% | 3.4% | 4.1% | 41.8% | 0.0% | 1.4% | 8.2% | 13.7% | 2.1% | | |

*ratio 2:1
